# The contribution of Medical Physics to Nuclear Medicine: looking back - a physicist’s perspective

**DOI:** 10.1186/2197-7364-1-2

**Published:** 2014-05-01

**Authors:** Brian F Hutton

**Affiliations:** Institute of Nuclear Medicine, University College London, Level 5 UCH, 235 Euston Road, London, NW1 2BU UK; Centre for Medical Radiation Physics, University of Wollongong, Wollongong, NSW 2522 Australia

**Keywords:** Nuclear Medicine, Physics, History

## Abstract

**Background:**

This paper is the first in a series of invited perspectives by four pioneers of Nuclear Medicine imaging and physics. A medical physicist and a Nuclear Medicine clinical specialist each take a backward look and a forward look at the contributions of Medical Physics to Nuclear Medicine.

**Discussion:**

Contributions of Medical Physics are presented from the early discovery of radioactivity, development of first imaging devices, computers and emission tomography to recent development of hybrid imaging.

**Summary:**

There is evidence of significant contribution of Medical Physics throughout the development of Nuclear Medicine.

## Background

Nuclear Medicine has its basis in ‘the use of radioactive tracers for medical diagnosis, therapy’. Since the inception of the discipline, the adoption of scientific methods and instruments has been central to its development and practice. Even today, the Nuclear Medicine service, at least in the larger tertiary medical centres and teaching hospitals, involves a team of individuals that includes scientists, clinicians, technologists, nurses and ancillary staff. The strength of Nuclear Medicine continues to be its unique sensitivity to picomolar concentrations of radiotracer that can be used to probe underlying physiological, biochemical and molecular processes. The exploitation of this capability continues to rely on joint ingenuity of scientific and clinical specialists.

The origins of Nuclear Medicine arose from the disciplines of both chemistry and physics (notably Marie Sklodowska Curie was awarded Nobel prizes in both fields). Chemistry was central to the discovery and production of radionuclides that are suitable for use in humans, and the development of specific radiolabelled compounds continues to challenge radiochemists and is central to evolving clinical practice. Equally important has been the development of instrumentation and image analysis tools for measurement and depiction of the *in vivo* radiotracer distribution that has involved a range of scientific disciplines. The focus in this article is on this latter group of scientists, collectively referred to as ‘medical physicists’, but including physicists, engineers, computer scientists, mathematicians and statisticians (including technologically oriented clinicians) who all contribute to the technological development that underpins Nuclear Medicine. A full review of the contributions of Medical Physics to Nuclear Medicine is well beyond the scope of this short article; instead, selected historical examples will be used to highlight the continuing significant clinical impact that medical physicists contribute to the field. Readers are referred to some general texts [1–4] and historical reviews [5–10] that provide more detailed description of technological developments in addition to selected key articles.

## Discussion

### Pre 1940: early pioneers

Clearly, the discovery of radioactivity by Henri Becquerel (1896) [11] and radium by Pierre and Marie Curie (1898) [12, 13] sets the scene for the whole discipline, and as early as 1913, the tracer principle was enunciated by Georg de Hevesy (a chemist) [14] leading to early use of radionuclides in humans by Blumgart and Weiss [15] and the first external radiation detectors. The invention of the cyclotron in 1932 by Ernest Lawrence (an engineer) [16], and subsequently the nuclear reactor, led very quickly to the production of useful radionuclides (e.g. iodine-131) [17] which were immediately used in early clinical studies [18]. Effectively, Nuclear Medicine was born.

### 1940 to 1960: from measurement to images

Early inventions of scintillation probes and counters as well as the Geiger counter continue to be used today in a range of applications, although limited in providing any spatial information (see [19]). They did however facilitate the development of dynamic studies to evaluate function and an early interest in understanding tracer dilution and uptake patterns, techniques that still permeate Nuclear Medicine practice, especially in the research setting. These formed the basis for clinical studies of renal function and cerebral blood flow. The invention of the rectilinear scanner by Benedict Cassen in 1951 [20], however, provided for the first time an image of the radiotracer distribution. This scanner in combination with *in vitro* studies and probe-based dynamic measurements provided the tools for early establishment of many Nuclear Medicine centres worldwide (in combination with use of radionuclides for therapy). But probably the invention that has had the highest impact on Nuclear Medicine practice is that of the gamma camera by Hal Anger in 1958 [21]. Detection of positron emission by coincidence detection using probes predated this [22, 23], but early imaging was mainly limited to single photon detection using either a rectilinear scanner or gamma camera.

### 1960 to 1980: computing and the birth of emission tomography

It took several years for the gamma camera to appear on the commercial market, and in the ensuing years, there was significant development of tomographic devices, initially based on probe systems but subsequently based on use of the basic gamma camera. David Kuhl is credited with development of the first tomographic systems (both emission and transmission) with reconstruction involving optical back projection without use of a computer [24, 25]. It should be remembered that computers were in their infancy at that time and researchers had to resort to innovative alternatives in order to reconstruct even crude images. It was not until the early 1970s [26] that computer-based reconstruction was introduced, very much influenced by the introduction of X-ray CT and availability of filtered back projection. In the meantime, a number of novel approaches were developed including focal plane (limited angle) tomography and early studies where the patient was rotated in front of a stationary camera [27]. The rotating gamma camera, the basis for most current single photon emission computed tomography (SPECT) systems, was first introduced by John Keyes in 1977 [28], with commercial demonstration by Ron Jaszczak around the same time [29].

The development of positron emission tomography (PET) followed a similar course with the earliest dual planar systems developed by Gordon Brownell at Mass General [30], and the first ring systems built in the early 1970s by Robertson, Yamamoto and colleagues at Brookhaven [31] and Michael Ter-Pogossian in conjunction with Mike Phelps and Ed Hoffman [32], who went on to install the first commercial system in 1976 and pioneer further early PET systems at UCLA. As with other sectors of Nuclear Medicine, it was the combination of chemistry (synthesis of FDG) and engineering/physics (development of medical cyclotrons and robust PET cameras) that triggered the widespread use of PET, initially as a research system but ultimately as an important clinical tool.

### 1980 to 2000: emission tomography comes of age

Both SPECT and PET became more widely available in this period of consolidation with both modalities being demonstrated to have health impact that justified their clinical use. During this period, early deficiencies were recognised and rectified and robust commercial systems developed. In the case of SPECT, this involved adoption of dual detectors and robust rotation gantries rather than the initial counter-balanced single head systems. PET systems benefitted from new technology (block detectors) initially using BGO and later LSO and LYSO. An important contributor to the efficacy of tomographic imaging was the development of iterative reconstruction based on maximum likelihood (MLEM) [33, 34], especially the accelerated form based on ordered subsets (OSEM) [35], which permitted computationally demanding algorithms to perform in a clinically acceptable time. The recent resurgence of time-of-flight measurement on systems using faster scintillators takes advantage of iterative reconstruction to provide signal-to-noise benefit.

The greater clinical demand for SPECT has also stimulated further novel systems specifically designed for a specific purpose (e.g. cardiac [36–38]). Previous focus on neurological systems proved less popular outside the research setting. These systems have taken advantage of detector technology (e.g. CZT) that has more recently been translated from high-energy physics laboratories, resulting in compact systems that have improved performance compared to the traditional gamma camera. These systems also offer potential for novel acquisition, adapting to the specific patient or study. As with most of the tomographic systems, developments in technology have driven the application, providing increasingly superior image quality and diagnostic capability.

### 2000 to present: hybrid imaging takes over

Both PET and SPECT benefit from attenuation correction based on direct estimation of the *in vivo* attenuation by means of transmission measurement [39, 40]. Developments of transmission systems for PET and SPECT were both based on external sources that could provide attenuation maps for correction. However, these were typically of poor quality, with noise in the transmission study propagating so as to deteriorate the emission study. Bruce Hasegawa initially suggested use of CT [41], leading to the introduction of a ‘low-cost, low-dose’ SPECT/CT in 1999 by GE Healthcare [42]. Subsequent developments have led to a range of fully diagnostic SPECT/CT systems. In the case of PET/CT, this was conceived initially in Geneva by David Townsend and colleagues, then developed at Pittsburgh (in conjunction with Ron Nutt at CTI) [43–45] and ultimately introduced commercially in 2000. The impact of PET/CT has been particularly impressive as the added diagnostic value of the combined modalities has by far exceeded expectations rather than simply providing efficient high-quality transmission measurement for the purpose of attenuation correction [46, 47].

The most recent hybrid imaging combination to be commercially released is PET/MRI, opening further potential for combining information and exploring joint synergies. This is another fine example of how innovative technology deriving outside the medical field (in this case, solid state readout systems that replace the photomultiplier of the conventional detector) can be adapted for benefit (magnetic compatibility, compact design, improved performance). The early developments in preclinical systems, e.g. [48], rapidly led to the introduction of paired sequential PET/MRI systems before the first demonstration of simultaneous PET/MRI in human neurological studies, quickly followed by whole-body applications [49, 50]. SPECT/MRI is not commercially available at this time, but development is in progress [51]; whether this proves clinically useful remains to be seen.

### Additional contributions

**Figure 1:**
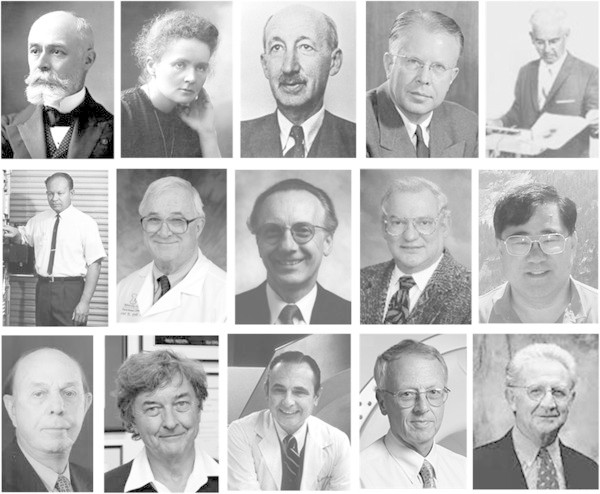
**Some of the many scientists who have contributed to Nuclear Medicine.** Top row (left to right): Henri Becquerel, Marie Sklodowska Curie, Georg de Hevesy, Ernest Lawrence and Benadict Cassen. Middle row: Hal Anger, David Kuhl, Gerd Muehllehner, Ron Jaszczak and Bruce Hasegawa. Bottom row: Gordon Brownell, Michael Phelps, Michael Ter-Pogossian, David Townsend and Ron Nutt.

The brief history above demonstrates very clearly how the growth of Nuclear Medicine can be attributed, at least in part, to the technological development. This history overlooks the associated research across the breadth of Nuclear Medicine that has led to the current practice. This includes international guidelines on radiation safety and dosimetry, accepted procedures for instrument specification, acceptance testing and quality control, and standards for digital image storage and transfer. The techniques used to guarantee image fidelity that are incorporated in commercial systems have been developed and validated (e.g. uniformity and linearity correction), the software for various image corrections necessary for quantification has evolved and continues to be developed (corrections for attenuation, scatter, resolution and motion), and image analysis software that facilitates quantification, image fusion, kinetic analysis and disease classification represents enormous international effort with significant impact on current clinical capabilities. The ‘medical physicist’ plays an important role as a member of the Nuclear Medicine team, providing scientific support in the clinical setting; equally, he/she continues to innovate and validate techniques that aid in improving the quality of information that can be extracted from medical images as well as the effectiveness of treatment. There are many active university-based research laboratories that continue to focus on the development of medical imaging instrumentation, image reconstruction and image analysis with many internationally recognised researchers contributing to the continuing development. It is impossible in this brief article to give adequate credit to the many individuals who have made major contributions to the field and have greatly influenced the tools that are available to current Nuclear Medicine practice (Figure 1). The many Medical Physics mentors and teachers should also not be overlooked as they have enabled the whole Nuclear Medicine community. The Medical Physics community also provides the manpower that populates the laboratories and support staff of the continually developing vendors.

## Summary

It is hard to overstate the value of the contribution of medical physicists to Nuclear Medicine. The development of the discipline has been driven by innovation from both scientists and clinicians. This partnership has been important in defining clinically relevant questions and seeking novel but practical solutions. Technology continues to develop (e.g. new detector materials and faster computers), and Medical Physics innovation continues to push the frontiers of what is possible. Effective translation from research laboratories to clinics requires specialised expertise and often lacks specific funding. Likewise, demonstration of clinical utility and impact is an important endeavour that requires dedicated staff or allocation of time, protected from normal clinical support demands. Medical physicists have been central to the development of Nuclear Medicine and must continue this essential work.
